# Circ_0078607 increases platinum drug sensitivity via miR-196b-5p/GAS7 axis in ovarian cancer

**DOI:** 10.1080/15592294.2023.2175565

**Published:** 2023-03-12

**Authors:** Cheng Dai, Shi-Yuan Dai, Yan Gao, Ting Yan, Qi-Yin Zhou, Shi-jun Liu, Xuan Liu, Dan-Ni Deng, Dong-Hong Wang, Qing-Feng Qin, Dan Zi

**Affiliations:** a Department of Gynecology, Guizhou Provincial People’s Hospital, Guiyang, P.R. China; b Department of Medical Record Statistics and Management, Guizhou Provincial People’s Hospital, Guiyang, P.R. China; c Department of Gynecology, the Second Affiliated Hospital of Zunyi Medical University of Guizhou Province, Zunyi, P.R. China; d Department of Gynecology, the Affiliated Hospital of Zunyi Medical University of Guizhou Province, Zunyi, P.R. China

**Keywords:** Circ_0078607, miR-196b-5p, GAS7, ovarian cancer, platinum resistance

## Abstract

Platinum-based chemotherapy is one of the predominant strategies for treating ovarian cancer (OC), however, platinum resistance greatly influences the therapeutic effect. Circular RNAs (circRNAs) have been found to participate in the pathogenesis of platinum resistance. Our aim was to explore the involvement of circ_0078607 in OC cell cisplatin (DDP) resistance and its potential mechanisms. Circ_0078607, miR-196b-5p, and growth arrest-specific 7 (GAS7) levels were assessed by qPCR. Circ_0078607 stability was assessed by ribonuclease R digestion and actinomycin D treatment. Cell viability of various conic of DDP treatment was measured by CCK-8. The cell proliferation was determined by CCK-8 and colony formation assay. Western blotting was performed for determining GAS7, ABCB1, CyclinD1 and Bcl-2 protein levels. The direct binding between miR-196b-5p and circ_0078607 or GAS7 was validated by dual-luciferase reporter and RIP assay. DDP resistance *in vivo* was evaluated in nude mice. Immunohistochemistry staining for detecting Ki67 expression in xenograft tumours. Circ_0078607 and GAS7 was down-regulated, but miR-196b-5p was up-regulated in OC samples and DDP-resistant cells. Overexpression of circ_0078607 inhibited DDP resistance, cell growth and induced apoptosis in DDP-resistant OC cells. Mechanistically, circ_0078607 sequestered miR-196b-5p to up-regulate GAS7. MiR-196b-5p mimics reversed circ_0078607 or GAS7 overexpression-mediated enhanced sensitivity. Finally, circ_0078607 improved the sensitivity of DDP *in vivo*. Circ_0078607 attenuates DDP resistance via miR-196b-5p/GAS7 axis, which highlights the therapeutic potential of circ_0078607 to counter DDP resistance in OC.

## Introduction

Ovarian cancer (OC) is the main cause of gynaecological-malignancy-related deaths worldwide [1]. Surgical resection combined with platinum-based chemotherapy remains the conventional treatment for OC. Platinum drugs can cause DNA crosslinks and DNA double-strand breaks in infinite proliferous cancer cells [2,3]. Although the platinum therapy has significantly improved the prognosis of OC, some patients have no response to platinum or suffer platinum resistance [4]. About 80% OC patients experience a recurrence within five years due to platinum resistance [5]. The lifecycle for OC patients with platinum resistance is within 12 months [6]. Currently, the molecular mechanisms of platinum resistance remain largely unknown. Therefore, uncovering the underlying mechanisms of platinum resistance is crucial to develop effective strategy for overcoming chemoresistance.

Circular RNAs (circRNAs) are a kind of closed single-stranded circular RNAs without coding potential. CircRNAs exert crucial regulation in multiple physiological and pathophysiological processes [7]. More importantly, the dysregulation of circRNAs has been shown to be implicated in chemoresistance. For instance, down-regulation of circRNA Cdr1as led to platinum drug resistance by modulating the miR-1270/SCAI axis in ovarian cancer [8]. Luo et al demonstrated the increased circFoxp1 level was responsible for cisplatin resistance in patients with OC [9]. Circ_0078607, derived from SLC22A3 gene, its expression has been demonstrated to be declined in OC and enforced circ_0078607 expression delayed OC development via miR-518a-5p/Fas pathway [10]. A recent study also reported that lower circ_0078607 level correlated with an adverse prognosis of high-grade OC patients [11]. However, whether circ_0078607 is involved in platinum resistance during OC is worth to be explored.

CircRNAs have been recognized to act as competitive endogenous RNAs (ceRNAs) to counteract the inhibitory functions of miRNAs in their target genes [12]. The aberrant expression of circRNA-miRNA-mRNA axis has been documented in various malignancies. CircFN1 functioned as miR-1205 sponge to promote E2F1 expression during sorafenib resistance in hepatocellular carcinoma [13]. Circ_0006528 facilitated paclitaxel resistance via sequestering miR-1299 to activate CDK8 in breast cancer [14]. Interestingly, both circ_0078607 and growth arrest-specific 7 (GAS7) were predicted to have putative binding sites in miR-196b-5p using bioinformatics analysis. MiR-196b-5p can act as a promoter of multiple tumours [15,16]. Repression of GAS7 resulted in enhanced metastatic potential of neuroblastoma [17]. In addition, miR-181a-mediated down-regulation of GAS7 contributed to gefitinib resistance in non-small cell lung cancer [18]. So far, the interaction among circ_0078607, miR-196b-5p and GAS7 during platinum resistance in OC has not been elucidated.

This article discovered the declined circ_0078607 level in OC tissues and cisplatin (DDP)-resistant OC cells. Functional studies indicated that overexpression of circ_0078607 sensitized OC cells to DDP via sponging miR-196b-5p to increase GAS7 expression. Uncovering the modulatory role of circ_0078607/miR-196b-5p/GAS7 axis provides an alternative method for reversing OC chemoresistance.

## Materials and methods

### Clinical samples

Forty-eight OC specimens and paired normal tissues were collected from patients diagnosed as OC through surgical resection at Guizhou Provincial People’s Hospital. All OC patients did not receive chemotherapy or radiotherapy before. This study was approved by the Ethics committee of Guizhou Provincial People’s Hospital. Written informed consents were obtained from all participants.

### Cell culture and treatment

OC cell lines (A2780 and SKOV3) were obtained from ATCC (Manassas, VA, USA) and normal ovarian epithelial cells (IOSE-80) were purchased from BioVector NTCC (Beijing, China). A2780 and SKOV3 cells were added with escalating concentrations of DDP to acquire DDP-resistant cells (A2780/DDP and SKOV3/DDP). These cells were cultured in RPMI 1640 medium (Thermo Fisher, Waltham, MA, USA) with 10% FBS (Thermo Fisher) under 5% CO_2_ at 37°C. To maintain the acquired DDP resistance, the culture media was supplemented with 0.3 μg/mL DDP.

### Cell transfection

For overexpression (oe), circ_0078607 or GAS7 sequences provided by GenePharma (Shanghai, China) were cloned into pcDNA3.1 to establish oe-circ_0078607 or oe-GAS7 plasmid. miR-196b-5p mimics and mimics negative control (NC) were acquired from GenePharma. All constructed plasmids or segments were transfected into A2780/DDP and SKOV3/DDP cells using Lipofectamine 2000 (Thermo Fisher).

### Quantitative polymerase chain reaction (qPCR)

Total RNA was extracted from A2780/DDP and SKOV3/DDP cells using the Total RNA Extractor (Sangon, Shanghai, China). For nuclear and cytoplasmic RNA separation, the Cytoplasmic and Nuclear RNA Purification Kit (Norgen Biotek, Thorold, Canada) was adopted. Then the Prime Script RT Master Mix (Thermo Fisher) was applied to generate cDNA. qPCR was performed using Universal SYBR Green Master Mix (Roche, Basel, Switzerland). The relative gene abundance normalized to GAPDH or U6 was analysed using 2^−ΔΔCt^ method. Primer sequences are as follows.

Circ_0078607 F: 5’-GACGCATTGCTAAGTGCAATGG-3’;

Circ_0078607 R: 5’- AGGTGAATGCTCCAGTCAGG-3’;

SLC22A3 F: 5’- GACGTGGATGACTTGCTACG −3’;

SLC22A3 R: 5’- GGCAATTCCAGGGAGAATTA −3’;

miR-196b-5p F: 5’- GGCGCTAGGTAGTTTCCTGTT −3’;

miR-196b-5p R: 5’- GCAGGGTCCGAGGTATTC −3’;

GAS7 F: 5’- CGAGCTACGTGCAGTTGCT −3’;

GAS7 R: 5’- CATGTGGGCAGTCTCTGGAG −3’;

U6 F: 5’- CTCGCTTCGGCAGCACATATACT-3’;

U6 R: 5’- ACGCTTCACGAATTTGCGTGTC-3’;

GAPDH F: 5’- CCAGGTGGTCTCCTCTGA-3’;

GAPDH R: 5’- GCTGTAGCCAAATCGTTGT-3’.

### Detection for circ_0078607 stability

For evaluation the stability of circ_0078607, the cells were treated with 2 mg/mL actinomycin D for 0, 8, 16, and 24 h before RNA extraction. In addition, the isolated RNA was digested with 3 U/μg ribonuclease R (RNase R) for 30 min at 37°C. After that, circ_0078607 and SLC22A3 levels were assessed by qPCR as described above.

### Cell counting kit-8 (CCK-8)

For cell viability detection, cells from different groups were exposed to various concentrations of DDP for 48 h and then incubated with 10 μL CCK-8 solution (Acmec Biochemical Co., Ltd, Shanghai, China) for 4 h. The results were detected at 450 nm on a microplate reader. As previously described, the cell viability of DDP treatment was analysed. For proliferation assay, CCK-8 (10 μL) was added to cells at the indicated time points. The following procedure was carried out as described above.

### Colony formation assay

Two hundred cells seeded in 6-well plate were maintained for 14 d in culture medium. After fixation in methanol, the colonies were immersed in 0.1% crystal violet solution. Finally, the colonies were photographed and quantified.

### Flow cytometry

The apoptosis of cells with multiple treatments was assessed using the Annexin V-FITC apoptosis assay kit (Absin, Shanghai, China). Briefly, the collected cells were stained with 5 μL Annexin V-FITC for 15 min in the dark. Then 5 μL PI solution was added to cells at 5 min before the detection on a flow cytometer (Partec, Germany).

### Western blotting

Total protein was acquired using cold RIPA lysis buffer (KEYGENE, Nanjing, China), followed by separation by SDS-PAGE and transferring onto polyvinylidene fluoride membranes. Blocking in 5% skim milk for 1 h was performed and then the membranes were probed with ABCB1 (A00049, 1:200, Boster, Wuhan, China), CyclinD1 (ab16663, 1:200, Abcam, Cambridge, UK), Bcl-2 (ab59348, 1:1000, Abcam), GAS7 (ab168370, 1:1000, Abcam) and GAPDH (A00227, 1:1000, Boster) at 4°C overnight. The secondary antibody was adopted for 30 min. The bands were displayed using ECL Substrates (Applygen, Beijing, China).

### Dual-luciferase reporter assay

The wild-type (WT) sequences of circ_0078607 and 3ʹ untranslated region (UTR) of GAS7 containing putative miR-196b-5p binding sites or their mutant (MUT) sequences were inserted into pmirGLO vector. Cells were transfected with the above plasmids together with miR-196b-5p mimics or mimics NC. After culture for 48 h, the relative luciferase activity was assessed using the Luc-Pair™ Duo-Luciferase Assay Kit (Yeasen, Shanghai, China).

### RNA immunoprecipitation (RIP) assay

RIP assay was performed using a Magna RNA-Binding Protein Immunoprecipitation Kit (Millipore). In short, A2780/DDP and SKOV3/DDP cells were lysed in RNA immunoprecipitation lysis buffer and probed with magnetic beads conjugated with anti-Ago2 (1:50, Millipore) or IgG (Millipore). After incubation with protease K, circ_0078607 and miR-196b-5p expression was evaluated by qPCR.

### Animal experiment

Twenty female BALB/c nude mice were obtained from Vital River Laboratory Animal Technology Co., Ltd (Beijing, China). A2780/DDP cells (1 × 10^7^) stably transfected with oe- circ_0078607 or oe-vector were subcutaneously injected into the mice. Seven days after the injection, DDP (3 mg/kg) or equal volume PBS was intravenously injected into the mice once a week. Tumour volume of each group was calculated every week. Five weeks later, the mice were sacrificed and tumours were removed for further analysis. All procedures were approved by the Ethics Committee of Guizhou Provincial People’s Hospital.

### Immunohistochemistry staining

The xenograft tumours were fixed in 4% formalin and embedded in paraffin. After deparaffinage, the tumour sections (5 μm) were received antigen retrieval and blocked in 5% goat serum. The primary antibodies against Ki67 (ab15580, 1:100, Abcam) was applied overnight at 4°C. After incubation with secondary antibody and DAB staining, the images were captured under a microscope. Using ImageJ software, the ki67 positive rate was calculated in five random fields. In each image, the ki67 positive cells and total number of cells per field were counted to determine the positive cell ratio.

### Statistical analysis

Data are presented as mean± standard deviation. GraphPad Prism was adopted for statistical analysis using Student’s t test or One-Way ANOVA. A p-value less than 0.05 was defined as statistically significant.

## Results

### Down-regulation of circ_0078607 in OC specimens and DDP-resistant OC cells

**Figure 1.**
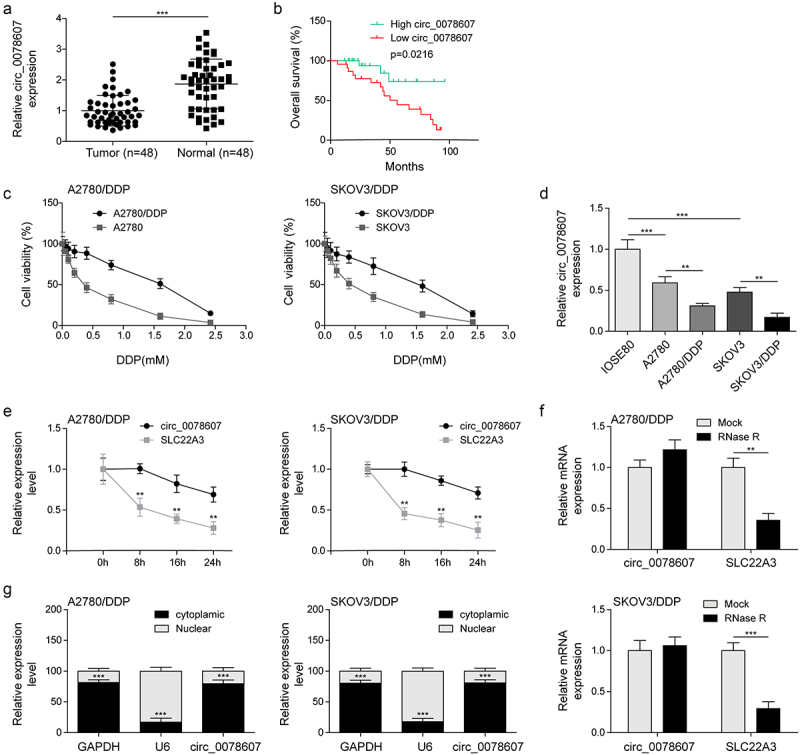
Decreased circ_0078607 abundance in OC tissues and DDP-resistant cells. (a) qPCR analysis of circ_0078607 level in OC clinical samples. (b) Kaplan-Meier survival plot of OC patients with high or low circ_0078607 level. (c) The cell viability at different concentrations of DDP treatment was detected by CCK-8. (d) qPCR analysis of circ_0078607 expressions in various cells. (e) Cells were exposed to actinomycin D for various time intervals, then circ_0078607 and SLC22A3 levels were assessed by qPCR. (f) The circ_0078607 and SLC22A3 levels in response to RNase R treatment were detected by qPCR. (g) qPCR for the nuclear and cytoplasmic expression of circ_0078607. **, P < 0.01; ***, P < 0.001.

To explore the function of circ_0078607 in DDP resistance, we firstly determined circ_0078607 abundance in OC specimens. As assessed by qPCR, circ_0078607 level in OC samples was obviously lower than control (Figure 1a). In addition, low circ_0078607 expression indicated a decrease in overall survival (Figure 1b) and was significantly associated with clinical stage and venous invasion (Table 1). DDP-resistant OC cells (A2780/DDP and SKOV3/DDP) were more resistant to various DDP concentrations (Figure 1c). Similarly, circ_0078607 expressions were declined in OC cells (A2780 and SKOV3) relative to normal IOSE-80 cells, which was further reduced by DDP resistance (Figure 1d). As shown in Figure 1e&F, circ_0078607 was resistant to actinomycin D or RNase R exposure, while linear SLC22A3 was degraded, suggesting the ultrastability of circ_0078607. Subsequently, circ_0078607 was mostly located in the cytoplasm of A2780/DDP and SKOV3/DDP cells (Figure 1g). Therefore, down-regulation of circ_0078607 might be involved in DDP resistance in OC.

**Table 1.** The clinicopathological parameters of patients with ovarian cancer.

	circ_0078607 expression RNAseq		
Parameters	High (n = 24)	Low (n = 24)	P value
Age			0.7702
≧60	15	13	
<60	9	11	
Clinical stage			0.0012
I/II	14	4	
III/IV	10	20	
Venous invasion			0.0189
No	15	7	
Yes	9	17	
Living Status			0.0003
Living	8	21	
Dead	16	3	

### MiR-196b-5p was a target of circ_0078607

**Figure 2.**
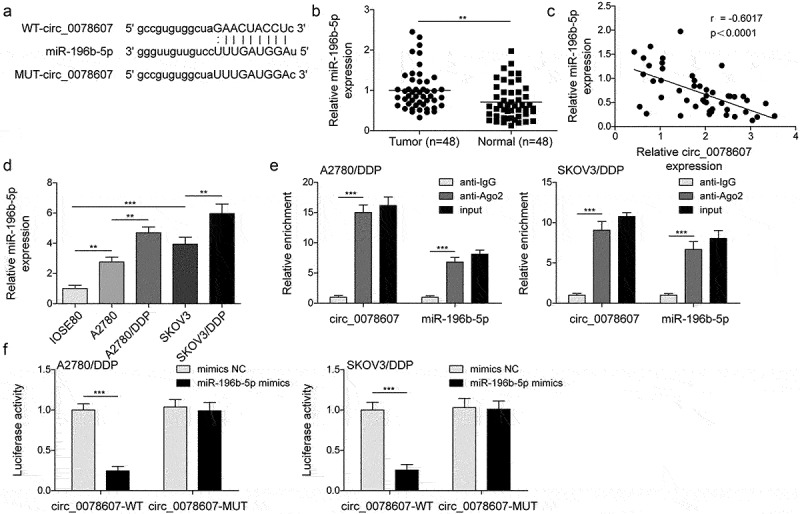
Circ_0078607 directly bond to miR-196b-5p. (a) The binding sites between circ_0078607 and miR-196b-5p. (b) qPCR for miR-196b-5p level in OC samples. (c) Pearson correlation analysis of circ_0078607 and miR-196b-5p expression. (d) qPCR for miR-196b-5p level in different cells. RIP assay (e) and dual-luciferase reporter assay (f) for the interaction between circ_0078607 and miR-196b-5p. **, P < 0.01; ***, P < 0.001.

As predicted by StarBase database, circ_0078607 possessed potential binding sites in miR-196b-5p (Figure 2a). Moreover, miR-196b-5p expression was demonstrated to be elevated in OC tissues (Figure 2b). We observed a negative correlation between circ_0078607 and miR-196b-5p (Figure 2c). Consistently, miR-196b-5p expression was elevated in OC cells, and further up-regulated in DDP-resistant cells (Figure 2d). Additionally, RIP assay confirmed the direct interaction between circ_0078607 and miR-196b-5p (Figure 2e). Besides, we found a remarkable decrease in luciferase activity after co-transfection with circ_0078607-WT and miR-196b-5p mimics, while miR-196b-5p mimics did not affect the circ_0078607-MUT, which further validated circ_0078607 directly interacted with miR-196b-5p (figure 2f). Collectively, circ_0078607 could directly sponge miR-196b-5p.

### Circ_0078607 increased DDP sensitivity by targeting miR-196b-5p

**Figure 3.**
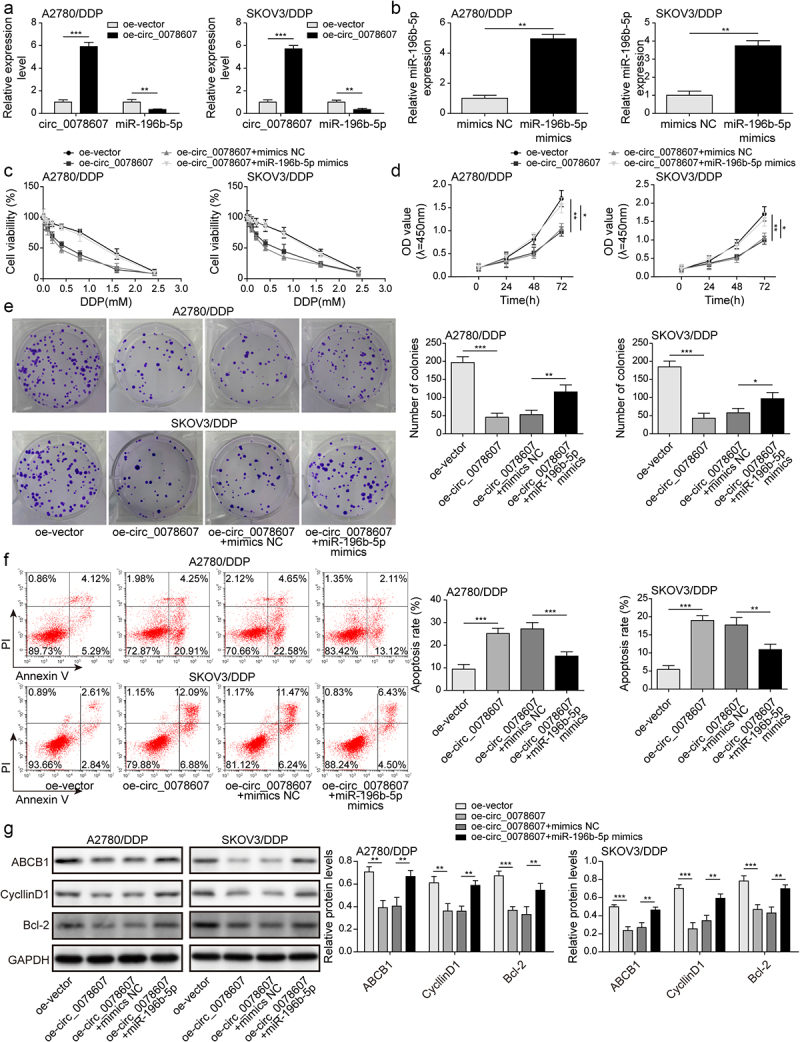
Circ_0078607 raised DDP sensitivity by sponging miR-196b-5p. (a) qPCR for circ_0078607 and miR-196b-5p expression after transfection with circ_0078607 overexpression plasmid. (b) MiR-196b-5p expression was determined by qPCR. (c) The cell viability at different concentrations of DDP treatment was detected by CCK-8. The proliferation was assessed by CCK-8 (d) and colony formation assay (e). (f) Flow cytometry for evaluating the apoptosis of cells. (g) Western blotting assay for determining ABCB1, CyclinD1 and Bcl-2 levels in A2780/DDP and SKOV3/DDP cells. *, P < 0.05; **, P < 0.01; ***, P < 0.001.

Given that circ_0078607 could directly interact with miR-196b-5p, we further determined whether circ_0078607/miR-196b-5p axis was implicated with DDP resistance of OC cells. Circ_0078607 overexpression led to a reduction in miR-196b-5p expression in A2780/DDP and SKOV3/DDP cells (Figure 3a). The enforced expression of miR-196b-5p was confirmed by qPCR (Figure 3b). Moreover, circ_0078607 overexpression evidently decreased the cell viability, which was overturned by miR-196b-5p mimics (Figure 3c). In addition, the growth of DDP-resistant cells was restrained by circ_0078607 overexpression, whereas miR-196b-5p overexpression abolished these changes (Figure 3d&E). Furthermore, enforced expression of circ_0078607 enhanced the apoptotic rate, which was partially reversed by co-transfection with miR-196b-5p mimics (figure 3f). Furthermore, ABCB1, CyclinD1, and Bcl-2 levels were declined in circ_0078607-overexpressed cells, however, miR-196b-5p mimics could partly restore the decreased expression of ABCB1, CyclinD1, and Bcl-2 (Figure 3g). Therefore, miR-196b-5p participated in circ_0078607-mediated enhanced DDP sensitivity.

### GAS7 was a target of miR-196b-5p

**Figure 4.**
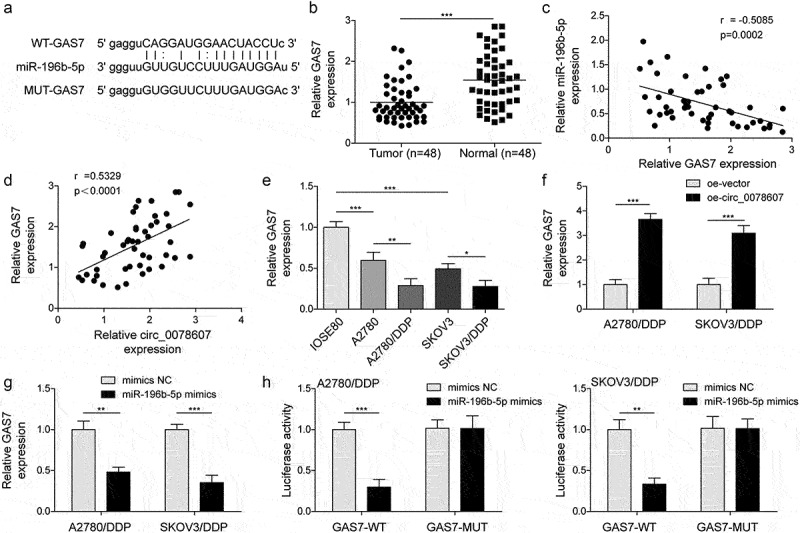
GAS7 was a down-stream target of miR-196b-5p. (a) The binding sites of miR-196b-5p in the 3’-UTR of GAS7. (b) qPCR for GAS7 mRNA expression in OC specimens. Pearson correlation analysis for evaluating the correlation between miR-196b-5p (c) circ_0078607 (d) and GAS7. (e) GAS7 mRNA expression in multiple cells was detected by qPCR. (f) qPCR for GAS7 abundance in circ_0078607-overexpressed cells. (g) GAS7 expression after transfection with miR-196b-5p mimics was assessed by qPCR. (h) The interaction between GAS7 and miR-196b-5p was determined by dual-luciferase reporter assay. *, P < 0.05; **, P < 0.01; ***, P < 0.001.

Next, the potential target of miR-196b-5p was analysed through StarBase database, and GAS7 was focused on. The potential binding sites between miR-196b-5p and GAS7 were illustrated in Figure 4a. In addition, a decreased expression of GAS7 was validated in OC samples (Figure 4b). Furthermore, GAS7 was negatively correlated with miR-196b-5p expression (Figure 4c), while positively correlated with circ_0078607 expression (Figure 4d). Besides, in comparison with normal IOSE-80 cells, GAS7 was down-regulated in OC cells. More importantly, GAS7 level was even lower in DDP-resistant OC cells (Figure 4e). In addition, GAS7 expression was enhanced by circ_0078607 overexpression (figure 4f), but reduced by miR-196b-5p mimics (Figure 4g). Additionally, miR-196b-5p mimics negatively regulated the luciferase activity of GAS7-WT, rather than GAS7-MUT (Figure 4h). These data suggested that miR-196b-5p could target GAS7 and repress its expression.

### MiR-196b-5p facilitated DDP resistance in OC cells by repressing GAS7 expression

**Figure 5.**
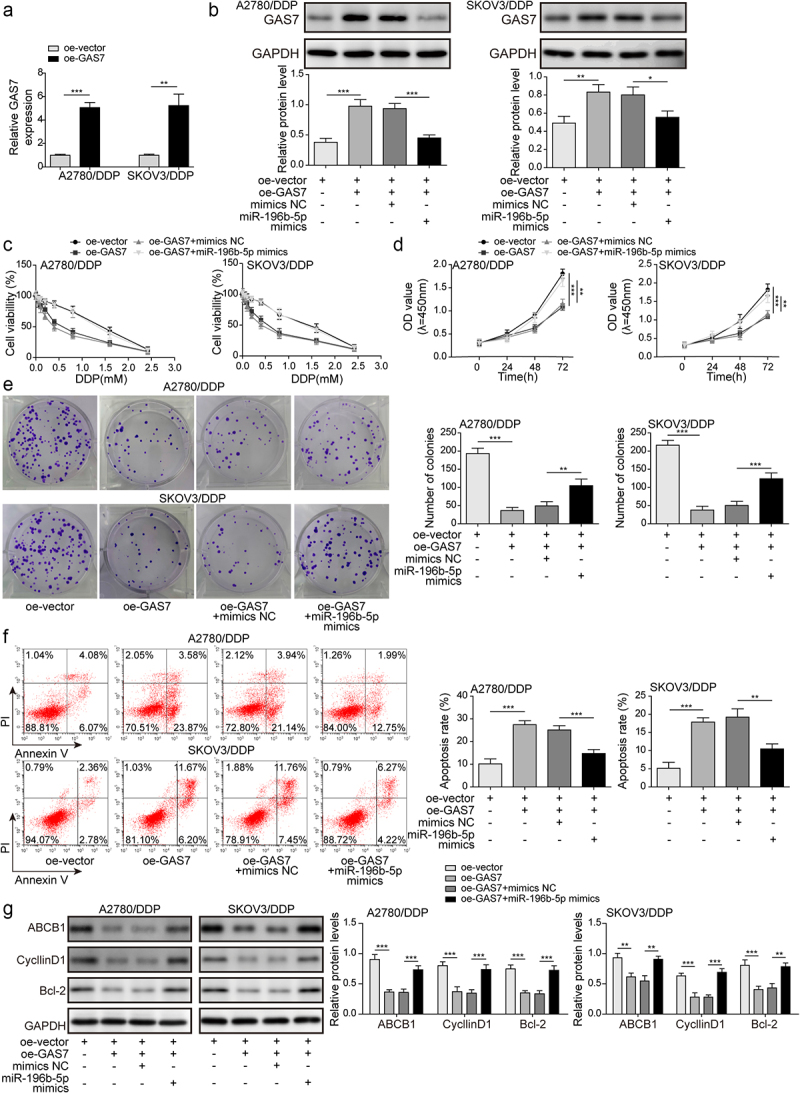
MiR-196b-5p conferred DDP resistance by targeting GAS7. (a) qPCR for GAS7 mRNA level after transfection with GAS7 overexpression plasmid. (b) Western blotting for evaluating GAS7 protein level in cells. (c) CCK-8 assay for determining cell viability at different concentrations of DDP treatment. (d) CCK-8 and (e) colony formation assays were used to detect the proliferation of cells. (f) Apoptosis was assessed by flow cytometry. (g) The protein levels of ABCB1, CyclinD1 and Bcl-2 were assessed by western blotting. *, P < 0.05; **, P < 0.01; ***, P < 0.001.

To further evaluate whether miR-196b-5p modulated DDP resistance of OC cells via targeting GAS7, cells were co-transfected with miR-196b-5p mimics and pcDNA3.1 GAS7 plasmid. Overexpression of GAS7 was validated by qPCR (Figure 5a). Western blotting further demonstrated that pcDNA3.1 GAS7-mediated overexpression of GAS7 was counteracted by miR-196b-5p mimics (Figure 5b). Subsequently, functional experiments indicated that overexpression of GAS7 decreased cell viability, inhibited proliferation, and induced apoptosis, whereas miR-196b-5p mimics could abolish pcDNA3.1 GAS7-mediated the above modulations (Figure 5c-f). In addition, we found declined ABCB1, CyclinD1, and Bcl-2 levels in GAS7-overexpressed cells, which were evidently abolished by miR-196b-5p mimics (Figure 5g). Thus, miR-196b-5p conferred DDP resistance in OC cells by targeting GAS7.

### *Circ_0078607 enhanced the sensitivity of DDP* in vivo

**Figure 6.**
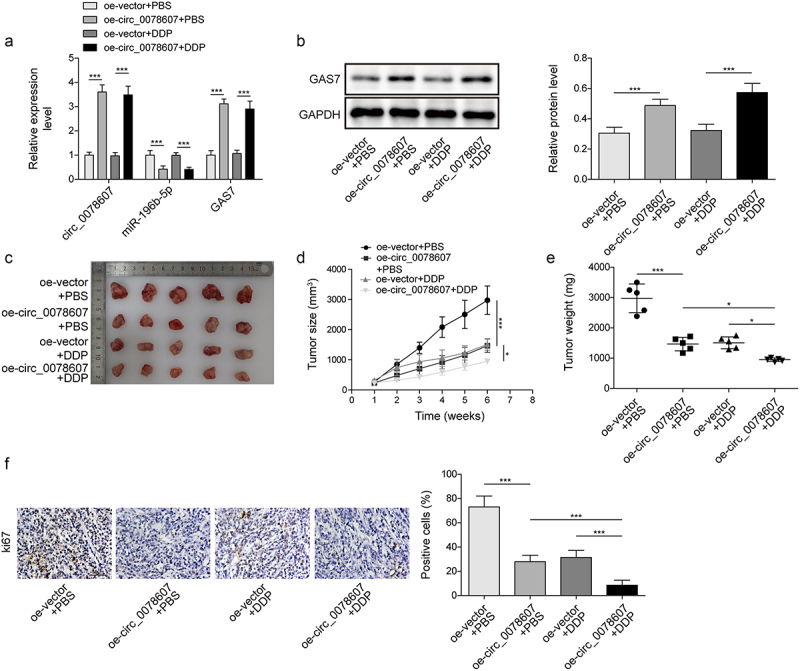
Circ_0078607 improved DDP sensitivity *in vivo*. (a) qPCR detected circ_0078607, miR-196b-5p, and GAS7 levels in tumour tissue. (b) Western blotting for evaluating the GAS7 protein level in tumour tissue. (c) The xenograft tumours of mice from different groups were shown. The growth curve (d) and weight (e) of subcutaneous xenograft tumours. (f) Immunohistochemistry staining for measuring Ki67 expression in xenograft tumours (200×). *, P < 0.05; ***, P < 0.001.

The regulatory functions of circ_0078607 in DDP resistance were further investigated in nude mice *in vivo*. In A2780/DDP cells implantation mice tumour tissues, we found that oe-circ 0078607 inhibited miR-196b-5p but promoted GAS7 expression, whereas DDP had no effect on these levels (Figure 6a-b). Furthermore, circ 0078607 overexpression or DDP administration reduced tumour volume and weight, which was more pronounced in the oe-circ 0078607 combination with DDP group (Figure 6c-e). Immunohistochemistry staining revealed a reduced Ki67 expression after overexpression of circ_0078607 or DDP treatment. Combined oe-circ_0078607 with DDP further reinforced this trend (figure 6f). Taken together, overexpression of circ_0078607 increased DDP sensitivity *in vivo*.

## Discussion

CircRNAs with prevalent expression in human tissues have been identified as regulators of a wide range of human diseases, including malignancy [19], Parkinson’s disease [20], acute kidney injury [21] and diabetes mellitus [22]. Recently, circ_0078607 has been reported to slow down OC development through miR-518a-5p/Fas pathway [10]. However, the biological regulation of circ_0078607 in OC chemoresistance remains obscure. Our data revealed that circ_0078607 was down-regulated in DDP-resistant OC cells. In addition, circ_0078607 alleviated DDP resistance via regulating miR-196b-5p/GAS7 axis. Our observations suggested circ_0078607 might be an effective target for treating DDP resistant OC.

During the last decades, chemotherapeutic drugs have substantially improved the survival of OC patients. DDP, one of platinum agents with broad-spectrum anticancer efficacy, has been widely used for the treatment of OC. Nevertheless, the resistance to DDP remains a key impediment for OC patients to achieve a satisfactory therapeutic efficacy [23]. To date, the roles of circRNAs in DDP resistance have been reported by limited studies [24,25]. In this study, circ_0078607 level was reduced in OC tissues and DDP-resistant OC cells. Next, rescue experiments suggested that overexpression of circ_0078607 reduced cell viability of DDP, restrained growth and induced apoptosis of A2780/DDP and SKOV3/DDP cells. Moreover, xenograft experiments indicated that ectopic expression of circ_0078607 enhanced the sensitivity of DDP *in vivo*. Therefore, loss of circ_0078607 might promote the progression of OC through conferring DDP resistance.

Next, we investigated the regulatory mechanisms of circ_0078607 underlying DDP resistance in OC cells. CircRNAs, distributed in cytoplasm, can function as ceRNAs of miRNAs to compete their activities during the development of different cancers [26]. It has been accepted that circRNAs possess miRNA binding sites, which sequester miRNAs to restrain miRNAs-mediated regulation in their target genes. For instance, circARNT2 facilitated DDP resistance in hepatocellular carcinoma cells through sequestering miR-155-5p to raise PDK1 expression [27]. miRNAs are involved in the development and chemoresistance of malignancies [28,29]. The oncogenic functions of miR-196b-5p have been reported in a variety of cancers, such as non-small cell lung cancer [16], colorectal cancer [15], lung adenocarcinoma [30], and so on. Herein, circ_0078607 was predicted to target miR-196b-5p by bioinformatics analysis. Moreover, we demonstrated the binding between circ_0078607 and miR-196b-5p. Furthermore, up-regulation of miR-196b-5p in OC samples and DDP resistant OC cells was negatively corelated with circ_0078607 level. More importantly, miR-196b-5p mimics reversed circ_0078607-mediated sensitivity to DDP in OC cells. These findings indicated that circ_0078607 affected DDP resistance of OC cells via direct binding to miR-196b-5p.

GAS7, as a growth arrest-specific gene, has been reported to hinder the progression of malignant tumours. A previous study indicated the tumour suppressive effect of GAS7 on acute myeloid leukaemia [31]. GAS7 depletion resulted in enhanced metastatic capacity of neuroblastoma [17]. Notably, GAS7 deficiency was implicated in miR-181a-mediated gefitinib resistance in non-small-cell lung cancer [18]. In our study, GAS7 was identified as a novel target of miR-196b-5p. As expected, GAS7 level was lower in OC specimens and DDP-resistant cells. circ_0078607 modulated GAS7 expression via sequestering miR-196b-5p. GAS7-mediated re-sensitivity of DDP could be antagonized by miR-196b-5p mimics. These data indicated that circ_0078607 could regulate DDP sensitivity through miR-196b-5p/GAS7 axis.

In conclusion, we demonstrated that circ_0078607 and GAS7 were down-regulated, while miR-196b-5p level was elevated in OC samples and DDP resistant cells. Further results indicated that circ_0078607 sensitized OC cells to DDP via sequestering miR-196b-5p, thereby reinforcing the expression of GAS7. Our observations provide a greater understanding of the elusive mechanisms of DDP resistance, identifying a novel potential treatment option to counter DDP resistance.

## Data Availability

All data generated or analyzed during this study are included in this article. The datasets used and/or analyzed during the current study are available from the corresponding author on reasonable request.
